# Exploring Histological Similarities Across Cancers From a Deep Learning Perspective

**DOI:** 10.3389/fonc.2022.842759

**Published:** 2022-03-30

**Authors:** Ashish Menon, Piyush Singh, P. K. Vinod, C. V. Jawahar

**Affiliations:** ^1^ Center for Visual Information Technology, International Institute of Information Technology (IIIT) Hyderabad, Hyderabad, India; ^2^ Center for Computational Natural Sciences and Bioinformatics, International Institute of Information Technology (IIIT) Hyderabad, Hyderabad, India

**Keywords:** TCGA, cross-organ inference, tissue morphology, class activation map (CAM), histopathology, deep learning, cancer classification

## Abstract

Histopathology image analysis is widely accepted as a gold standard for cancer diagnosis. The Cancer Genome Atlas (TCGA) contains large repositories of histopathology whole slide images spanning several organs and subtypes. However, not much work has gone into analyzing all the organs and subtypes and their similarities. Our work attempts to bridge this gap by training deep learning models to classify cancer vs. normal patches for 11 subtypes spanning seven organs (9,792 tissue slides) to achieve high classification performance. We used these models to investigate their performances in the test set of other organs (cross-organ inference). We found that every model had a good cross-organ inference accuracy when tested on breast, colorectal, and liver cancers. Further, high accuracy is observed between models trained on the cancer subtypes originating from the same organ (kidney and lung). We also validated these performances by showing the separability of cancer and normal samples in a high-dimensional feature space. We further hypothesized that the high cross-organ inferences are due to shared tumor morphologies among organs. We validated the hypothesis by showing the overlap in the Gradient-weighted Class Activation Mapping (GradCAM) visualizations and similarities in the distributions of nuclei features present within the high-attention regions.

## 1 Introduction

Cancers originating from different organs and cell types are known, with the most common ones being breast, lung, colorectal, prostate, and stomach. The most common causes of cancer deaths are lung, colorectal, and liver (1). Pan-cancer omics studies have revealed commonalities in driver mutations, altered pathways, and immune signatures (2, 3). Molecular profiling helps to cluster and distinguish different cancers and their subtypes by different computational methods (4–7). Given the diverse nature of different cancers and their origin, it will also be interesting to examine the morphological patterns that are unique and shared across different cancers from the histopathological standpoint. Histopathology continues to play a crucial role in cancer diagnostics. Digitization of tissue samples as whole slide images (WSIs) enables computer-based diagnosis and analysis. The deep learning approaches can be used to analyze the cancerous and non-cancerous patterns present in these tissues.

Deep learning has significantly improved the accuracy of a wide variety of computer vision tasks. The success of convolutional neural networks (CNNs) in the ImageNet Large Scale Visual Recognition Competition (8) resulted in a widespread adoption of CNNs for the task of image recognition, object detection, and image retrieval in several fields. Different studies show the effectiveness of CNNs and the utility of models with ImageNet pretrained weights in analyzing the tissue (9–14). Coudary et al. (12) extracted 512 *×* 512 non-overlapping patches of whole slide tissue images as input image patches for the WSI. The method rejected all the background and noisy patches with a mean intensity of half of the pixels greater than a set threshold. An ImageNet pretrained Inception-v3 (15) network was finetuned for the classification of cancerous and non-cancerous lung tissue slides. Tabibu et al. (11) extended the same idea to the renal cell carcinomas and performed cancer vs. normal classification and subtype classification by finetuning the entire ResNet-18,34 (16) networks and reported both slide-wise and patch-wise results. Wang et al. (13) adopted a threshold-based segmentation for background region detection by operating on the Hue Saturation Value (HSV) color space to get the required mask for patch filtering and identified the regions of metastatic breast cancer using ImageNet pretrained GoogLeNet (17). Xu et al. (10) performed classification and segmentation tasks on brain and colon pathological images using CNNs for feature extraction and training using a fully connected network (FCN). There are also few attempts to perform pan-cancer analysis using a deep learning approach. Fu et al. (18) have used features from models trained for cancer vs. normal classification task to predict genomic, molecular, and prognostic associations across organs. Cheerla et al. (19) have used multimodal learning to predict survival from genetic data as well as histopathology images across organs. Noorbakhsh et al. (20) have reported the correlation of organs based on the slide-wise area under the receiver operating characteristic curve (ROC-AUC). In this work, training is performed at the patch level, and inference is made at the slide level using a threshold for the fraction of patches in a slide predicted as cancerous. They used inception v3 (15) by using the CNN as a feature extractor and finetuning the last fully connected layer. They also performed hierarchical clustering of slide-wise ROC-AUC scores across organs and showed correlations of logits of the models of specific organs to suggest shared tumor morphology. We took this a step further to analyze cross-organ correlations quantitatively as well as qualitatively.

The contribution of this work is three-fold:

- Analyze each slide at the patch level and report high patch-level cancer vs. normal accuracies to set high benchmarks.
- Reveal tumor similarities between certain groups of organs/subtypes using patch-level analysis of WSIs from a deep learning perspective.
- Demonstrate the consistencies of these correlations both qualitatively and quantitatively, which is the first of its kind to our knowledge.

We reported the self organ classification results with AUC, F1 score, and accuracies for 11 cancer subtypes and the best and worst cross-organ inference results for each of these trained models.

In the cross-organ inference, the trained models are used for inference on the images of the other organs. The t-distributed stochastic neighbor embedding (t-SNE) (21) plot of embeddings obtained from each trained model shows the separability of cancer and normal features across organs. The GradCAM visualization of each trained model tested on the patches of other organs supports the cross-organ performance between a specific pair of organs, indicating the presence of common morphological patterns. We showed that the distributions of the nucleus features present in the high-attention regions for pairs with good cross-organ performance are well aligned compared to those with poor cross-organ performance. A uniform workflow which performs satisfactorily across organs is established. This includes patch extraction from tissue-rich regions of WSI based on intensity values and connected components present in its binarized format, hyperparameter tuning (using Bayesian optimization) to decide on the model architecture.

## 2 Method

### 2.1 Dataset and Preprocessing

**Figure 1:**
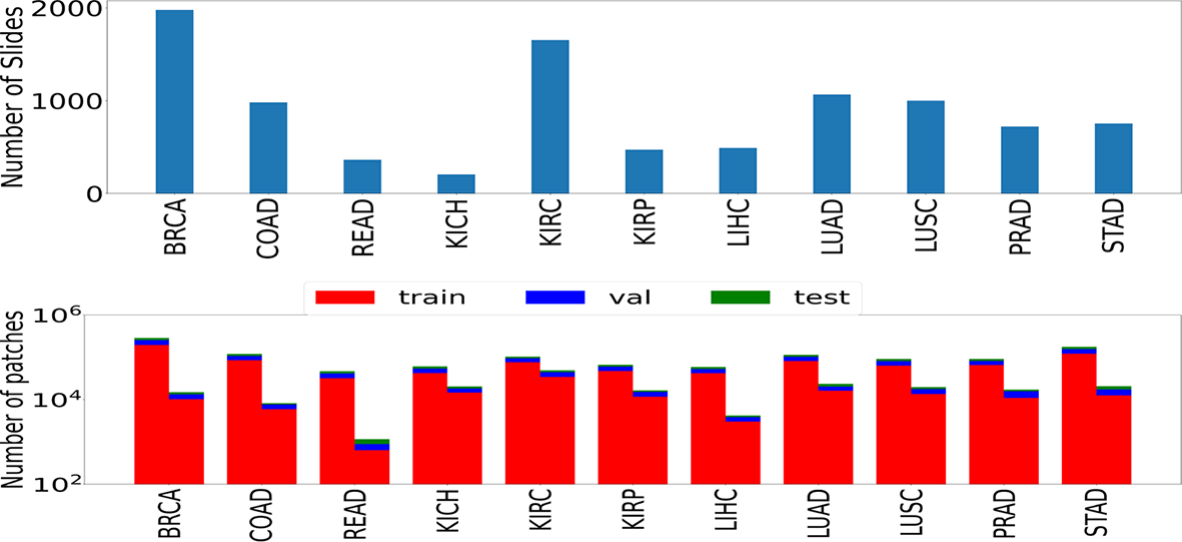
The number of slides (top) and patches (bottom) used in the study. Numbers of patches belonging to both classes (left bar represents cancer samples and right bar represents normal samples) are shown in the form of two rectangular bar plots.

We used the publicly available data set of WSIs from TCGA project (22) across multiple organs. Experiments were performed using the formalin-fixed paraffin-embedded (FFPE) slides. As pointed out by (23), the FFPE sections reveal useful cellular details of the tissue. These slides can confirm the diagnosis, in contrast to the frozen slides that can affect the morphological features of the tissue. 9,792 whole slide images spanning seven organs, namely, breast, colorectal, kidney, liver, lung, prostate, and stomach, were used. Some of these organs have multiple subtypes: lung [lung adenocarcinoma (LUAD) and lung squamous cell carcinoma (LUSC)], kidney [kidney renal clear cell carcinoma (KIRC), kidney renal papillary cell carcinoma (KIRP), and kidney chromophobe (KICH)], and colorectal [colon adenocarcinoma (COAD) and rectum adenocarcinoma (READ)]. We also considered cancer images specific to breast [breast invasive carcinoma (BRCA)], stomach [stomach adenocarcinoma (STAD)], liver [liver hepatocellular carcinoma (LIHC)], and prostate [prostate adenocarcinoma (PRAD)]. The number of slides and images considered in this study are shown in **Figure 1**.

H&E-stained WSI contains several cells and comprises as many as tens of billions of pixels, which is computationally infeasible for training neural networks. Resizing the entire image to a smaller size would hamper the cellular-level details, resulting in lower classification performance (24). Therefore, the entire WSI is commonly divided into partial patches or tiles analyzed independently. We adopted the strategy mentioned in Coudary et al. (12), by extracting 512 *×* 512-sized patches with no overlap at a ×20 magnification. The patch-filtering method of (11) was used to filter out background and noisy patches. We also added another patch-filtering step to avoid patches with a fractal structure by considering only those patches with ten or more connected components present in its binarized format. Since patch-wise labels were not available for TCGA dataset, the slide label was assigned to patches as shown to be effective by (11, 12). A train-validation-test split of 70–20*–*10 was performed before training the models. Data augmentation techniques such as random horizontal flip and random crop were used to improve generalizability. The images were normalized using the mean and standard deviation across all the three (RGB) channels calculated on the training set.

### 2.2 Cancer vs. Normal Classification

**Figure 2:**
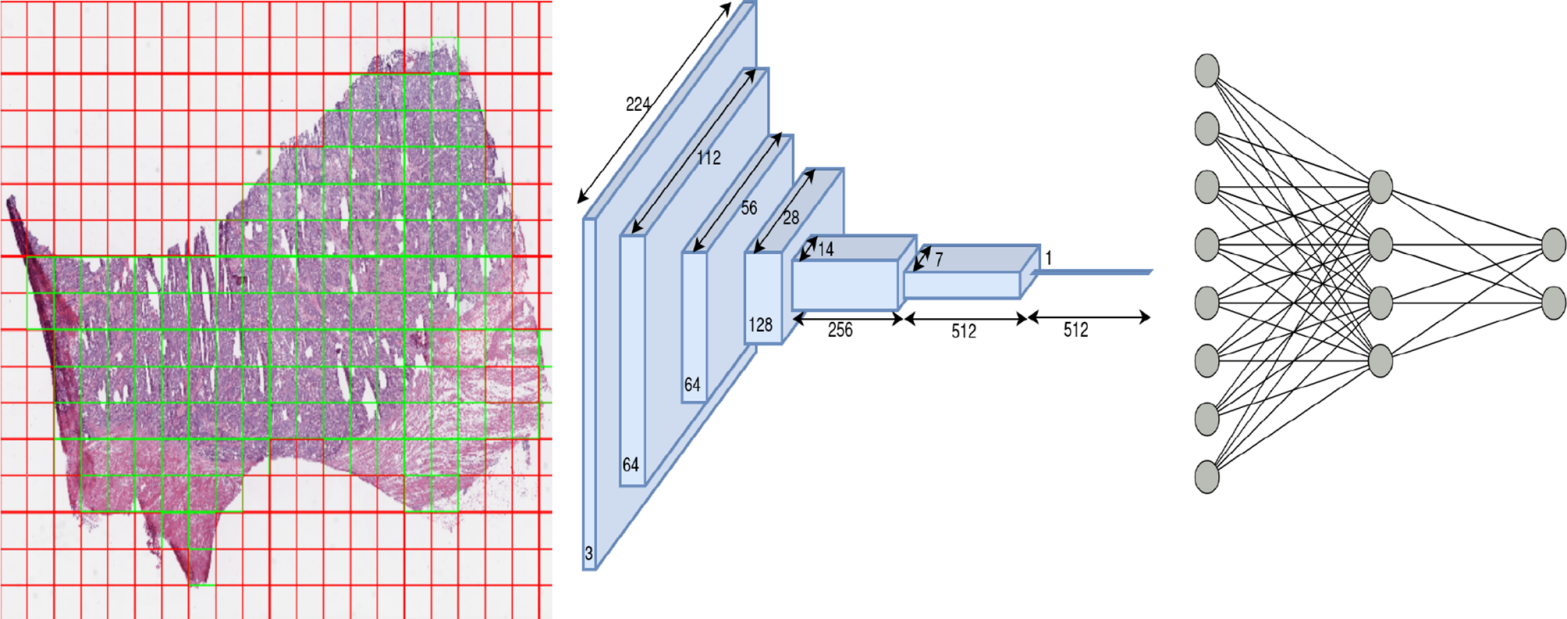
Overview of architecture used in our work: patch extraction (left): red shows rejected background patches, and green shows patches used for the training model, ResNet-18 architecture (middle) and Fully connected network (right).

We trained one model for each of the eleven subtypes (eleven models in total) using a ResNet-18 architecture pretrained on the ImageNet dataset. The ResNet style of architecture has performed well compared to other computer vision models on the ImageNet dataset (16). ResNet-18 was chosen over other models (ResNet-34,50,101) since 18 layers were found sufficient to yield superior performance in the classification tasks across most cancers, and a further increase in the number of layers led to a marginal increase in performance at the expense of a large increase in the number of trainable parameters. The schematic flow diagram is shown in **Figure 2** for the classification task. We replaced the last layer of ResNet-18 which provided the logits for the thousand classes of the ImageNet classification task with a fully connected network (FCN). The size of the last layer of this FCN was fixed at two since the task was a binary classification.

The entire network parameters were optimized to minimize the cross-entropy loss on the train data *via* backpropagation. The optimizer, learning rate, number of FCN layers, number of neurons in each layer, and dropout probabilities for each FCN layer were chosen by a hyperparameter search using Bayesian optimization. The batch size was set to 256. Owing to the class imbalance in the cancer and normal samples across organs, weighted cross entropy was used as the loss function. We also employed a stratified sampling technique to maintain the ratio of positives and negatives.

### 2.3 Hyperparameter Search

We used Optuna framework (25) for hyperparameter tuning with the search space of the optimizer sampled from a categorical distribution of optimizers (Adam, RMSProp, SGD), learning rate sampled from a log-uniform distribution of values ranging [1*e−*05, 1*e−*01], dropout sampled from a uniform distribution of values from [0.2, 0.5], number of layers of FCN uniformly sampled from values [1, 3], and number of neurons per layer uniformly sampled from values ranging [4, 128]. We ran 20 trials for hyperparameter search, and in each trial we trained the model for 20 epochs. Finally, the optimal hyperparameters that had the maximum validation accuracy across all trials were used to train the model for 50 epochs. We tested the usefulness of hyperparameter tuning on four organs and found a significant improvement in the performance (accuracy, AUC, F1 score). Hence, we adopted the same strategy for all the other organs during the training. The contour plot indicating the hyperparameter tuning is shown in **Supplementary Figure S1**.

### 2.4 GradCAM Analysis

We used the GradCAM (26) visualization technique to support the cross-organ inference results. We obtained a thresholded GradCAM heatmap and a bounding box over the high-attention region for each of the patches under study. Thresholding of the high-attention regions (green) of the heatmap was done by converting the image to the HSV color space, since the hue channel models the color type and is helpful in segmenting regions based on a specific color criteria. To obtain the bounding box containing the segmented region, we applied canny edge detection to the thresholded image. For each of the obtained contours, we applied closed-polygon approximation followed by finding a rectangular bounding box. We explored through these thresholded and bounding box outputs whether the regions of high saliency have overlap across models trained on different organs. We quantified the overlap by using IoU (intersection over union) of the bounding box representations, with IoU = 1 representing a perfect overlap and IoU = 0 representing no overlap. We also reported the Jaccard index to quantify the overlap using the thresholded pixel maps.

### 2.5 Nucleus Feature Extraction

**Figure 3:**
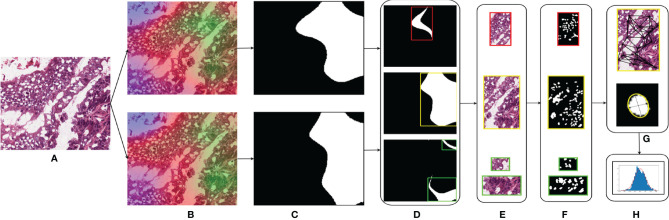
Nucleus segmentation workflow involved in segmenting nuclei from the specific regions of a sample patch: **(A)** COAD sample patch, **(B)** GradCAM outputs of BRCA model (top) and COAD model (bottom), **(C)** thresholded GradCAM mask, **(D)** BRCA-specific mask (top), overlapping mask (middle), and COAD-specific mask (bottom), **(E)** masked regions of BRCA-specific (top), overlap (middle), and COAD-specific (bottom), **(F)** nucleus segmented regions of BRCA-specific (top), overlap (middle), and COAD-specific (bottom), **(G)** obtaining the nucleus shape and graphical features for each region, and **(H)** distributions of these features.

Different studies have demonstrated the association of nucleus features to the clinical outcome and molecular data (11, 27–29). We hypothesized that the shared regions between cancers might show similar nucleus shapes and density features due to the similarity in the tumor microenvironment. We used the GradCAM high-attention regions to analyze the geometrical features of the nuclei such as eccentricity, convex area, region solidity, diameter, major axis, and minor axis and graphical features such as Voronoi diagram, Delaunay triangulation, minimum spanning tree, and nucleus density that characterize the arrangement of nuclei. We compared the distributions of these features to comment on the shared tumor morphology. The steps involved are shown in **Figure 3**.

- Region extraction: for the patches under study, we first extracted the high-attention regions corresponding to the model trained using that organ and the high-attention regions of the model trained on the other organ. We extracted three regions, the overlapped area of intersection and areas specific to each of the models. The overlap region was obtained by performing a logical AND operation between the thresholded GradCAM images. Specific regions were obtained by subtracting the overlapped regions from the thresholded GradCAM images.
- Nucleus segmentation: for each of the extracted regions, we performed the nucleus segmentation using a hierarchical multilevel thresholding approach (30).
- Nucleus features: we extracted geometrical shape features from the nucleus segmented images using the connected component analysis (11). Inter-nucleus architecture-based features were obtained by using graph-based techniques (31).

## 3 Results and Discussion

### 3.1 Quantitative Analysis

**Figure 4:**
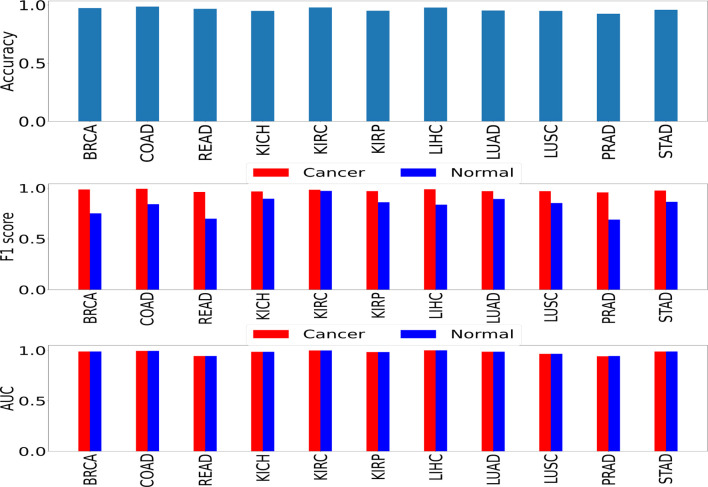
Self-organ inference showing the performance obtained using models trained on each cancer and tested on a held-out test set of the same cancer.

**Figure 5:**
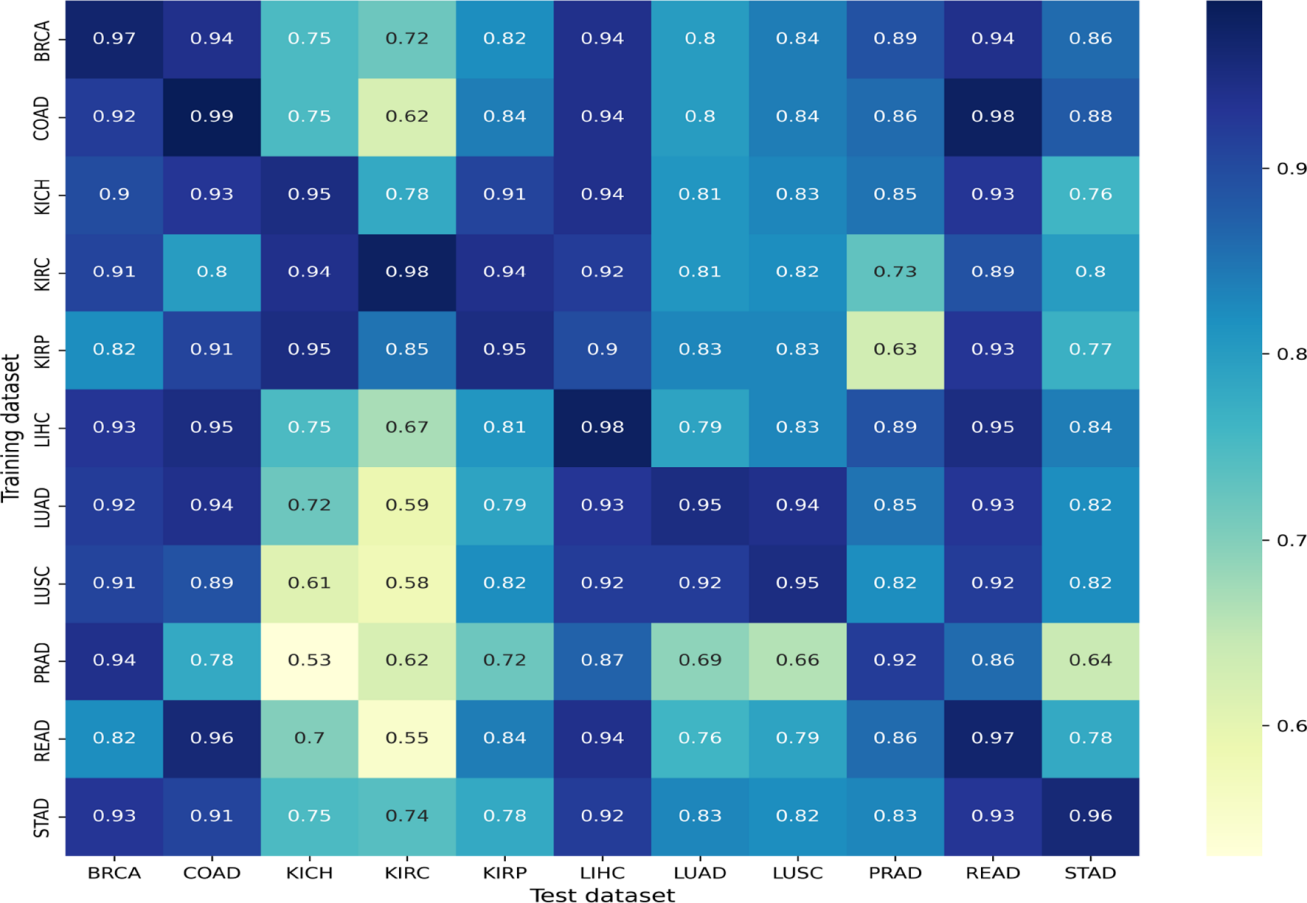
Cross-organ inference results: accuracies obtained using models trained on the organs along the rows and tested on the organs along the column are shown.

We performed two sets of experiments for the overall analysis. The first experiment was to come up with a trained model for the cancer vs. normal classification task in each of the mentioned organs/subtypes. A high classification performance was observed for most models (**Figure 4**). The second experiment was the cross-organ inference by testing each of these trained models on the held-out test of all the other organs. We report similarities between specific organ pairs based on performance (accuracy *>* 0.9) (**Figure 5**). Best and worst performances (AUC, F1) for the cross-organ inference are indicated in **Table 1**. The ROC curve for the cross-organ inference is shown in **Figure S2**.

**Table 1:** Cross-organ inference indicating the quantitative results of best and worst inferences of individually trained models when tested on other unseen organs.

Model	F1 score				AUC			
	Best		Worst		Best		Worst	
BRCA	READ	0.9443	KICH	0.6600	READ	0.9837	KIRC	0.7815
COAD	READ	0.9799	KIRC	0.5287	READ	0.9981	KIRC	0.6246
KICH	COAD	0.9294	STAD	0.7519	KIRP	0.9783	STAD	0.8163
KIRC	KIRP	0.9423	PRAD	0.7678	KICH	0.9881	PRAD	0.8069
KIRP	KICH	0.9490	PRAD	0.6893	READ	0.9840	PRAD	0.6692
LIHC	READ	0.9442	KIRC	0.6157	READ	0.9893	KICH	0.7203
LUAD	LUSC	0.9381	KIRC	0.5675	LUSC	0.9831	KIRC	0.6256
LUSC	BRCA	0.9251	KIRC	0.5769	LUAD	0.9683	KIRC	0.5998
PRAD	BRCA	0.9422	KICH	0.5632	LIHC	0.9453	KICH	0.5481
READ	COAD	0.9680	KIRC	0.4987	LIHC	0.9507	KIRC	0.5246
STAD	READ	0.9410	KICH	0.6921	BRCA	0.9822	KICH	0.8062

### 3.2 Cross-Organ Similarities

We found that most models show a good cross-organ inference accuracy when tested on BRCA, LIHC, COAD, and READ (**Figure 5**), which suggests that these cancers may have shared tumor morphologies. Colorectal subtypes (READ and COAD) show similarities with each other along with BRCA and LIHC. These observations on COAD, READ, and BRCA are consistent with the clustering of pan-gynecological and pan-gastrointestinal observed by (20). In contrast, most of the models perform poorly when tested on the kidney (KIRC, KIRP, and KICH) and lung subtypes (LUAD and LUSC). This suggests that kidney and lung cancer subtypes have morphology features localized relative to the organ of origin. The unique characteristics of kidney cancers are also seen with respect to their gene expression pattern as observed in our previous work (32). Interestingly, within cancer subtypes, we also observed that the performance of KICH and KIRP models on KIRC as a test set does not yield comparable performance. This suggests that KIRC has more subtype-specific features that are not present in other subtypes. Although READ and STAD are gastrointestinal cancers, the cross-organ inference is not high using the READ model. We observed that the cross-organ performance is not uniform within adenocarcinomas (LUAD, COAD, PRAD, READ, and STAD).

**Figure 6:**
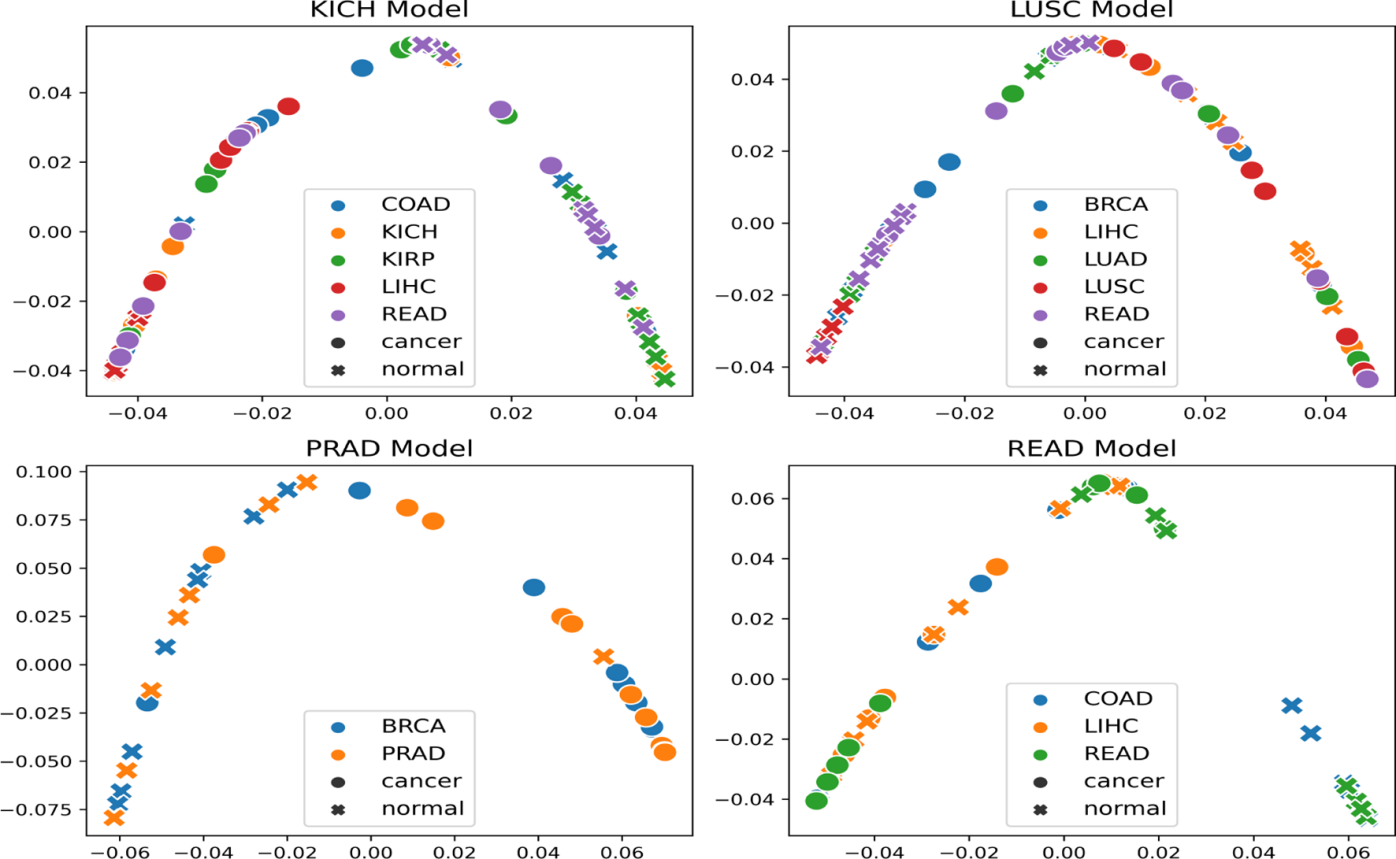
t-SNE embeddings of the trained models (mentioned in the title of each figure) helping to visualize the separability of cancer and normal embeddings of organs unseen by the trained models.

The t-SNE embedding was obtained for different model-organ pairs. **Figure 6** shows t-SNE plots for KICH, LUSC, PRAD, and READ. The t-SNE plots of other model-organ pairs are shown in the supplementary section (**Figure S3**).

The embeddings show that the models are able to exhibit separability in feature space between cancer and normal patches for the subtype that it was trained on as well as for subtypes/organs with cross inference accuracy >90%. However, the t-SNE embeddings also indicate that few of the normal and cancer samples are at close proximities after projection to the 2D space. This could possibly be attributed to the models not being fully accurate, the 2D projection error, or the assumption that all patches in a cancer slide are cancerous.

### 3.3 Cross-Organ GradCAM Visualization

**Figure 7:**
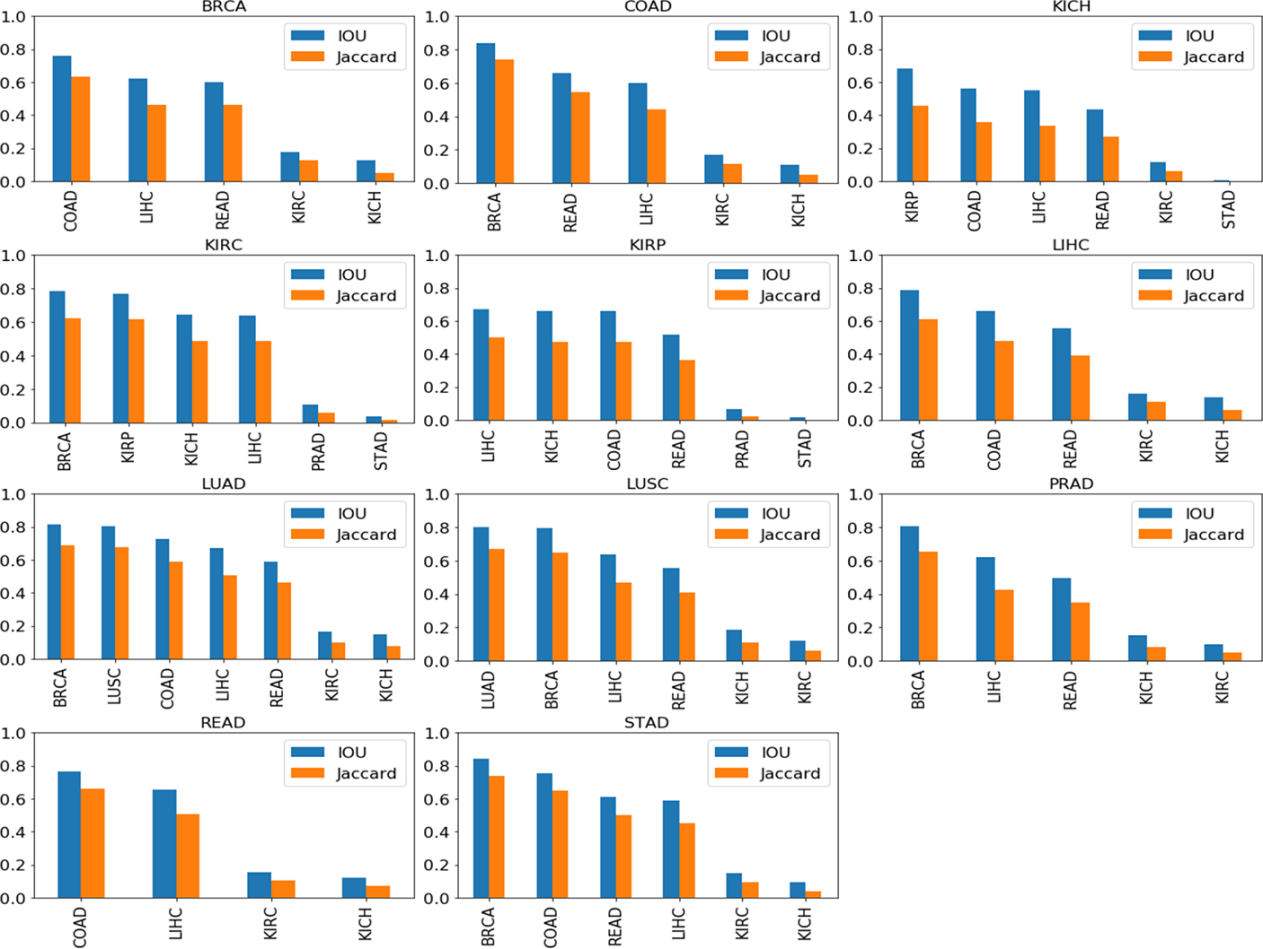
Cross-organ GradCAM results showing the IoU and Jaccard index of high-attention regions. The model used for visualization is indicated on the title of each plot, and the subtypes used are indicated on the x-axis.

A further qualitative analysis was done comparing the GradCAM outputs of the model-organ pairs, with cross-organ inference accuracy >90% as well as cross-organ inference accuracy *<* 80%. **Figure 7** shows the quantitative results of the degree of overlap between GradCAM outputs using the IoU and Jaccard index.

**Figure 8:**
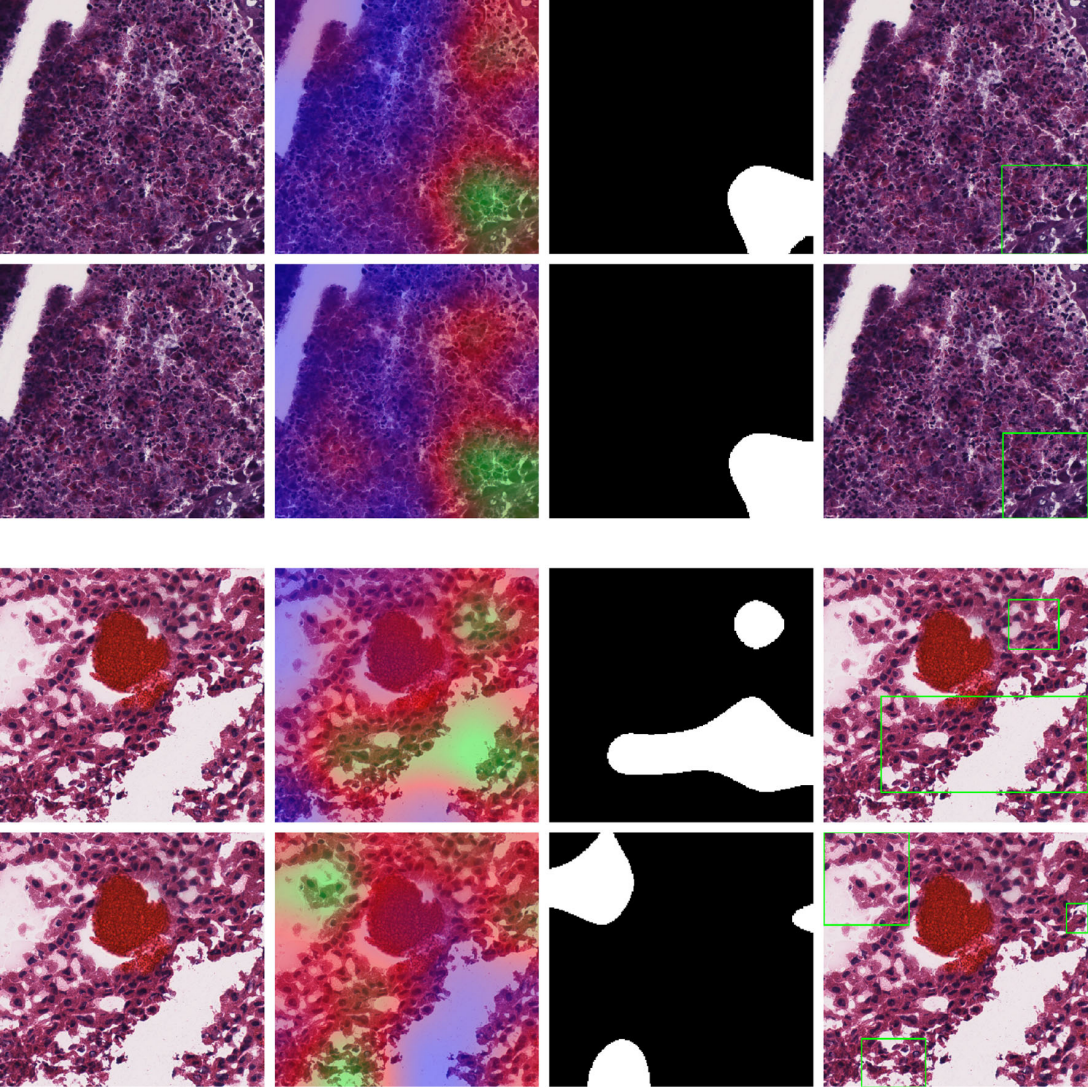
Cross-organ GradCAM visualization of the BRCA model on COAD and KICH cancer patches. Columns show the input patch, GradCAM output, GradCAM thresholded, and GradCAM with bounding box, respectively. Top 2 rows show COAD input patches and visualization using the BRCA model (1st row) and COAD model (2nd row). Bottom 2 rows show KICH input patches and visualization using the BRCA model (3rd row) and KICH model (4th row).

**Figure 8** shows the visualization using the BRCA model on COAD, LIHC, and READ subtypes. The visualization for other cross-organ inferences are provided in the supplementary section (**Figures S4**, **S5**). The visualization outputs in green indicate regions with high attention, those in red indicate regions with moderate attention, and those in blue indicate no attention during the classification task. Ground-truth visualizations for the patch of an organ are obtained by using the model trained on the same organ. We compared the degree of overlap of the visualization outputs to comment on the shared tumor morphology. We observed a positive correlation between the observed cross-organ inference accuracy, i.e., the IoU and the Jaccard index are high for model-organ pairs with high cross-organ inference accuracy and low for model-organ pairs with low cross-organ inference accuracy. For example, the BRCA model has the highest cross-organ accuracy, highest IoU, and Jaccard index on COAD. The same trend is observed in the models of other organs.

### 3.4 Cross-Organ Similarities Seen in the Distribution of Nucleus Features

**Figure 9:**
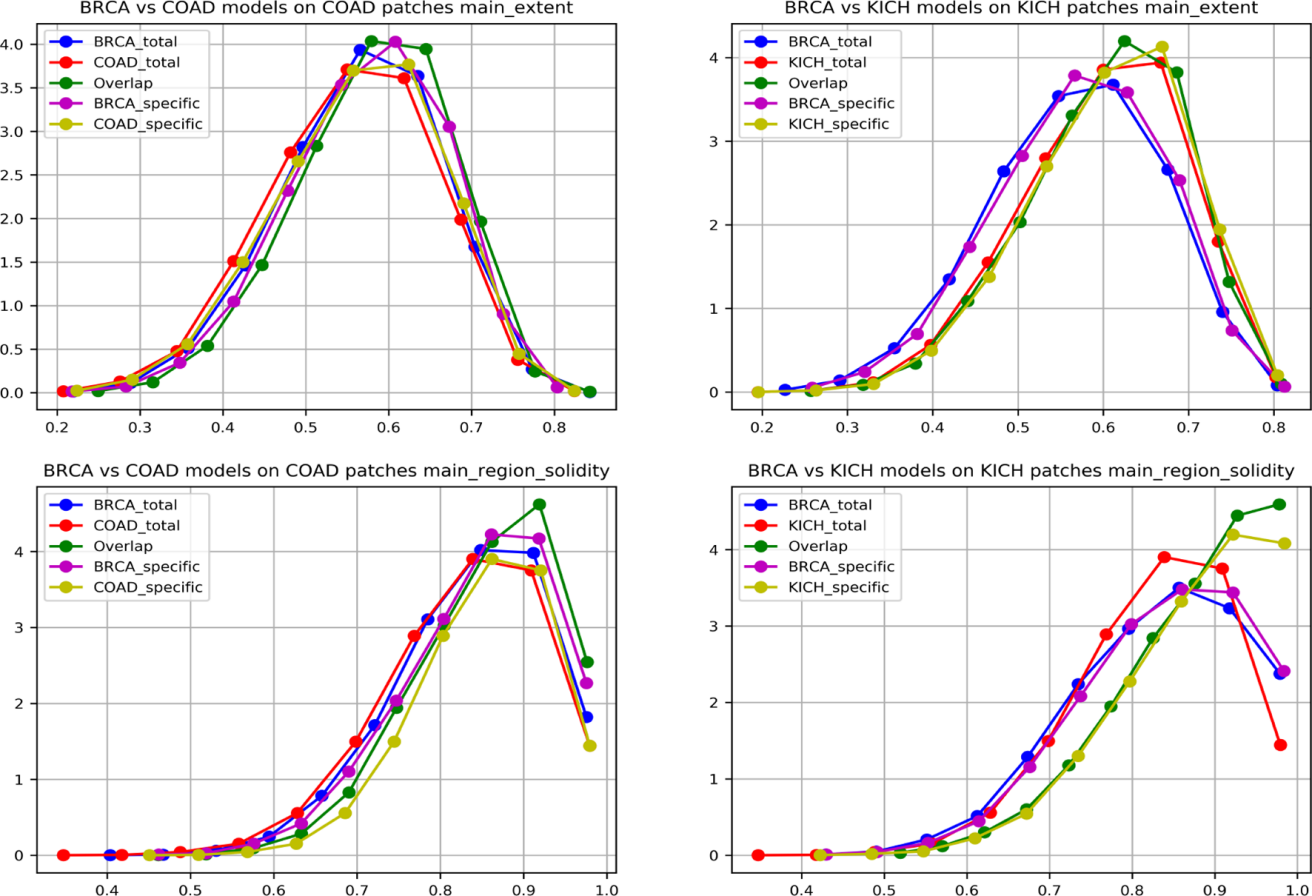
Graph showing nuclei shape distribution of BRCA and COAD models inferred on COAD patches (left) and BRCA and KICH models inferred on KICH patches (right). The x-axis represents value of the feature, and the y-axis represents the PDF. In each subplot, “Total” is the overall high-attention region of the corresponding model, “overlap” is the common region of high attention for the two models, and “specific” is the “total” region excluding the “overlap”.

To further strengthen the hypothesis about cross-organ similarities, we observed the distribution of shape features of the nuclei present in the high-attention regions. We considered two groups that showed good (BRCA and COAD) and another that showed poor (BRCA and KICH) performances in cross-organ inferences to characterize the nucleus morphological characteristics. We considered the high-probability patches [*P* (*cancer*) *>* 0.98] of COAD and KICH for the analysis. The distributions of some of the geometrical features of nuclei (main region extent and solidity) present in the regions focused by BRCA and COAD models on COAD patches are similar and correlated in contrast to the distributions seen with BRCA and KICH model on KICH patches (**Figure 9**).

We found that eight nucleus shape features and three inter-nucleus density features are significantly (p-value greater than 0.05) associated with the similarities observed between tumor morphologies (**Table 2**). Some of the significant nucleus shape features include total area (p-value = 0.0736), main extent (p-value = 0.1002), main region solidity (p-value = 0.0583), and some of the significant nucleus density features include neighbor count within a radius of 10, 20, and 30 pixels (p-value = 0.5974, 0.6044, 0.1945). We observe from the cross-organ performance table and the cross-organ GradCAM results that the BRCA model performs well on COAD patches and poorly on KICH patches and a similar behavior is seen in the distribution of nucleus geometrical features observed between the pairs of two groups (BRCA-COAD and BRCA-KICH).

**Table 2:** Nucleus feature statistical analysis: the table showing the p-values obtained after performing the t-test on two pairs of groups (BRCA-COAD) and (BRCA-KICH).

Feature type	Features	p-value (BRCA and COAD)	p-value (BRCA and KICH)
Nucleus shape features	Total area	0.0736	1.0711E-132
	Total convex area	0.0823	4.6482E-147
	Total perimeter	0.0004	3.3526E-240
	Total filled area	0.0738	9.6456E-133
	Total major axis	0.0004	3.3976E-228
	Total minor axis	0.0002	1.8502E-194
	Total peri by area	0.0768	1.9164E-235
	Main region area	0.0171	2.0913E-246
	Main region convex area	0.0182	1.1829E-228
	Main region eccentricity	0.1387	4.3698E-18
	Main extent	0.1002	5.0677E-37
	Main region solidity	0.0583	2.4308E-34
	Main region perimeter	0.0007	0
	Main region angle	0.091	0.4033
	Main region peri by area	0.0142	1.4962E-203
	Main region major axis	0.0004	0
	Main region minor axis	0.0014	0
	Total diameter	0.0003	7.524E-225
Inter-nucleus density features	Neighbor count within a 10-pixel radius	0.5974	0.0009
	Neighbor count within a 20-pixel radius	0.6044	6.6978E-07
	Neighbor count within a 30-pixel radius	0.1945	1.0807E-19

A higher p-value indicates similarity, and a lower p-value indicates differences. The high p-values of the BRCA-COAD group go in agreement with the observed distribution plot.

## 4 Conclusion

In this work, we explored tumor features and morphology across multiple organs from a deep learning perspective. This has not been extensively studied compared to the pan-cancer studies based on molecular profiling. We report similarities based on very high performance obtained with models trained on one cancer and tested directly on another. This level of performance can be achieved only if the learnt features are general or common between cancers. Our observations span not only cancers originating from the same organ but also different organs, which are interesting. We observed that good cross-organ performance is also reflected in the separability of normal and cancerous patches in feature space when visualized using the t-SNE plot.

We also explored GradCAM techniques to establish that the models with high cross inference accuracy had a significant overlap in their attention regions. This suggests that the deep learning model is able to pick up shared morphological features that span across organs during classification. We further showed similarity at the nucleus level by analyzing the distribution of geometrical and graphical features of nuclei present in the overlapping and non-overlapping regions. Overall, our study presents the proof-of-principle experiment that deep learning and computational approaches can be adopted to explore the shared morphology across different cancers. There is a need for further characterization at the experimental level, which will be taken up as future work. We made publicly available the model checkpoints, the source code, and the best model architectures for most common cancers using TCGA data. All the resources can be accessed from the project page at https://bhasha.iiit.ac.in/tcga_cross_organ_project.

## Data Availability Statement

Publicly available datasets were analyzed in this study. These data can be found here: https://portal.gdc.cancer.gov/.

## Ethics Statement

Ethical review and approval were not required for the study on human participants in accordance with the local legislation and institutional requirements. Written informed consent for participation was not required for this study in accordance with the national legislation and the institutional requirements.

## Author Contributions

Conceptualization, AM, PS, PV, and CJ. Methodology, AM and PS. Software, AM and PS. Validation, PV and CJ. Formal analysis, AM. Investigation, PS. Resources, AM and PS. Data curation, PS. Writing—original draft preparation, AM and PS. Writing—review and editing, PV and CJ. Visualization, AM, PS. Supervision, PV and CJ. Project administration, CJ. Funding acquisition, PV and CJ. All authors contributed to the article and approved the submitted version.

## Funding

We thank IHub-Data, International Institute of Information and Technology, Hyderabad, for the financial support.

## Conflict of Interest

The authors declare that the research was conducted in the absence of any commercial or financial relationships that could be construed as a potential conflict of interest.

## Publisher’s Note

All claims expressed in this article are solely those of the authors and do not necessarily represent those of their affiliated organizations, or those of the publisher, the editors and the reviewers. Any product that may be evaluated in this article, or claim that may be made by its manufacturer, is not guaranteed or endorsed by the publisher.
