# Supplementary material for: Exploring Histological Similarities Across Cancers From a Deep Learning Perspective
Source: Front Oncol. 2022 Mar 30;12:842759. doi: 10.3389/fonc.2022.842759 (PMC9006948; doi:10.3389/fonc.2022.842759)
Supplement: Supplementary file 1 [file DataSheet_1.pdf]

## 9. Supplementary Results

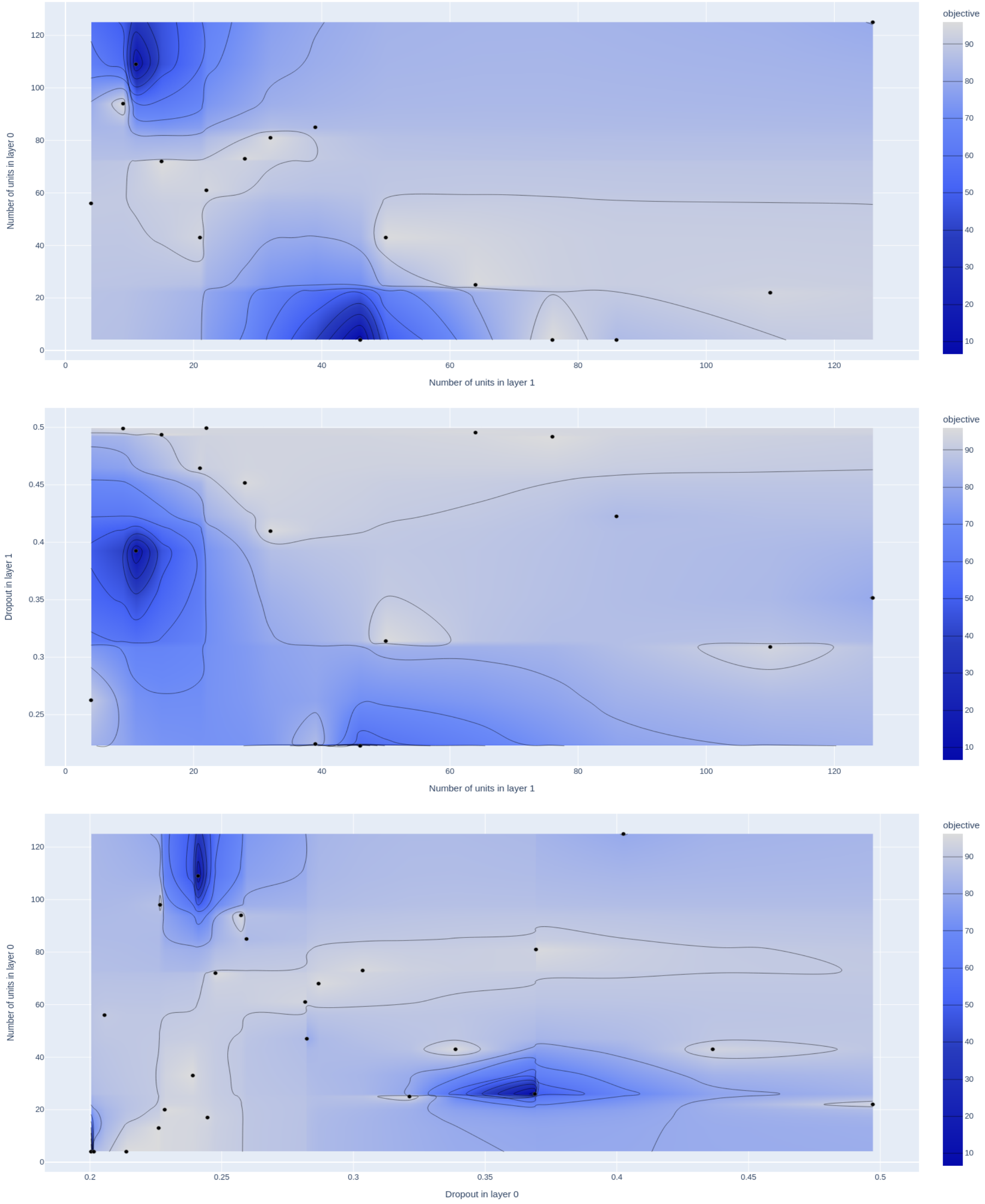


Figure S1: Contour plots from hyperparameter tuning process for BRCA. In each subplot X and Y axes show the hyperparameters and color denotes objective (validation accuracy).

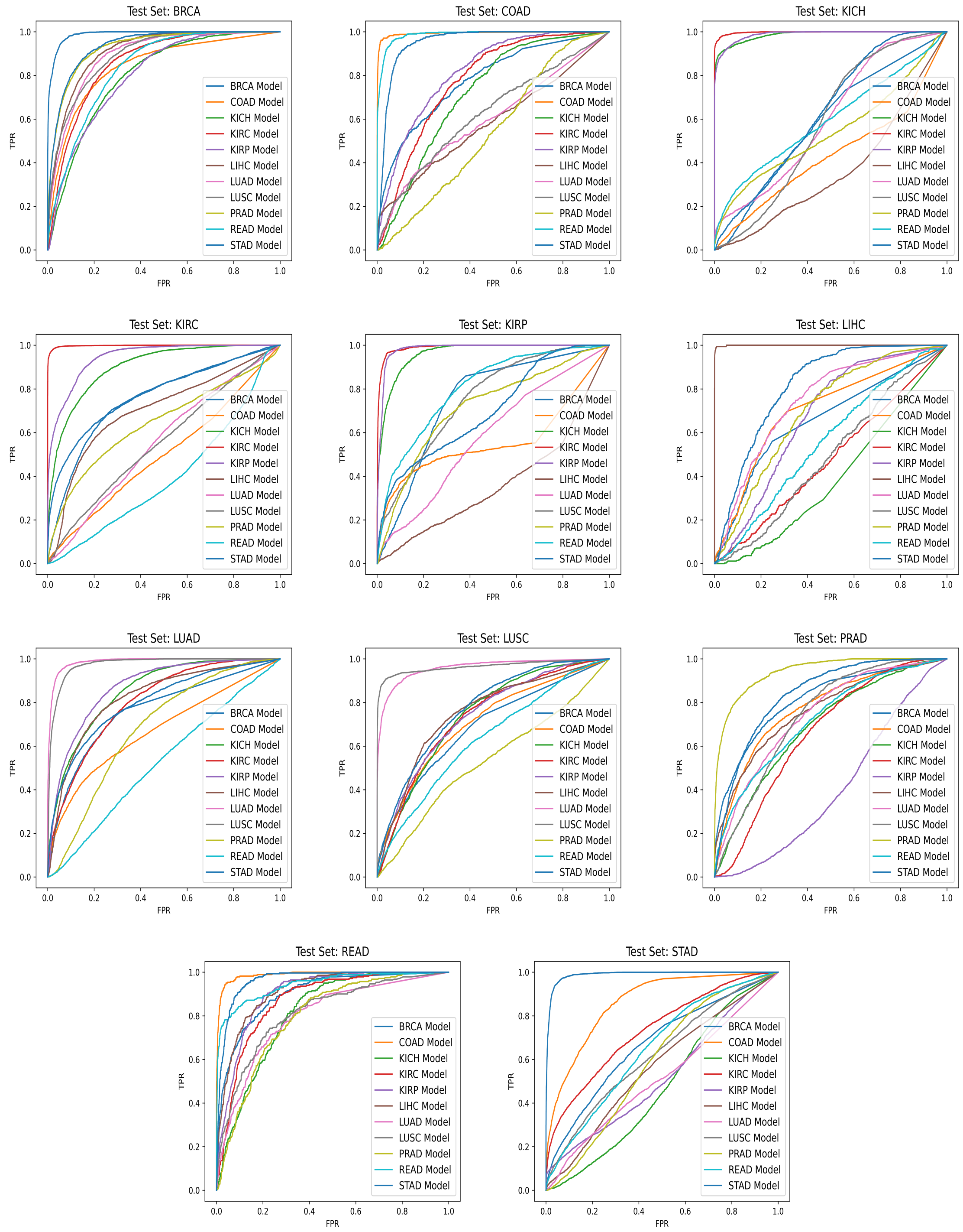


Figure S2: **ROC curves**: performance of each trained model tested across all the organs by considering normal to be positive class and cancer to be negative class.

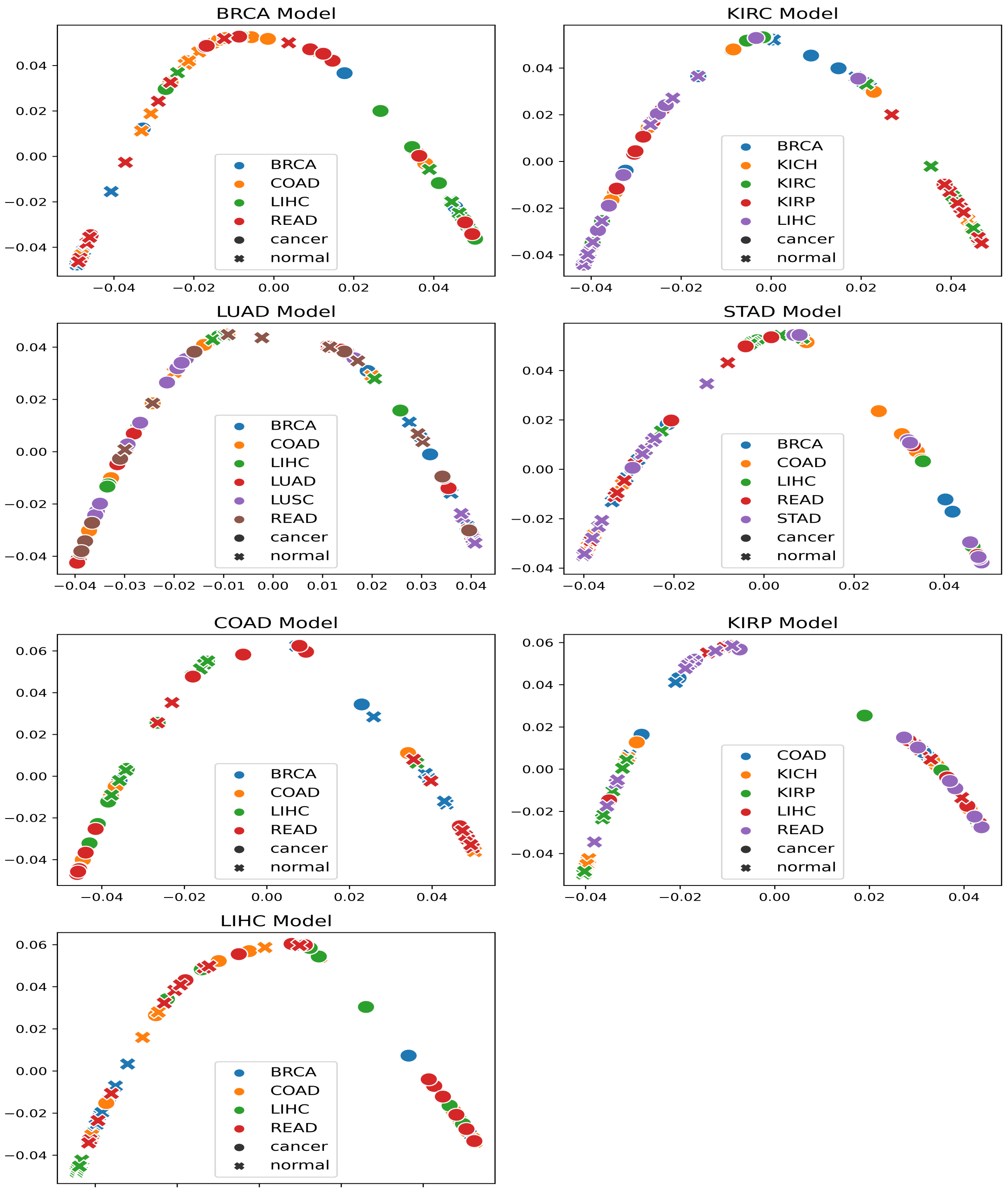


Figure S3: t-SNE plots of embeddings obtained from model trained on the organs indicated in the title of each plot when tested on the patches of organs indicated in the inset.

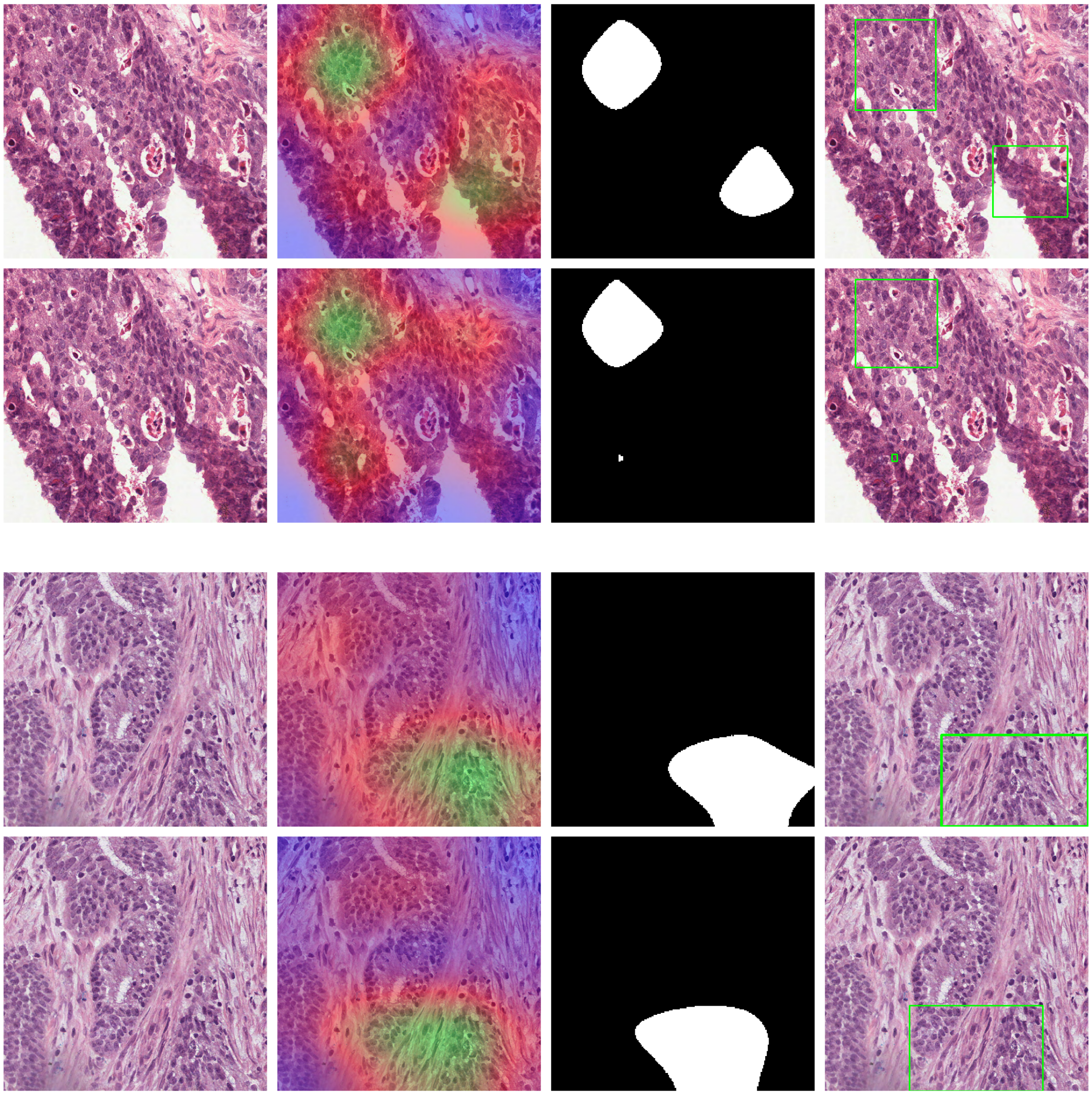

Figure S4: Cross-organ GradCAM visualization: First and the second row show visualization of COAD and READ model respectively on a sample READ patch. Third and the fourth row shows visualization of LUAD and COAD model respectively on a sample COAD patch.

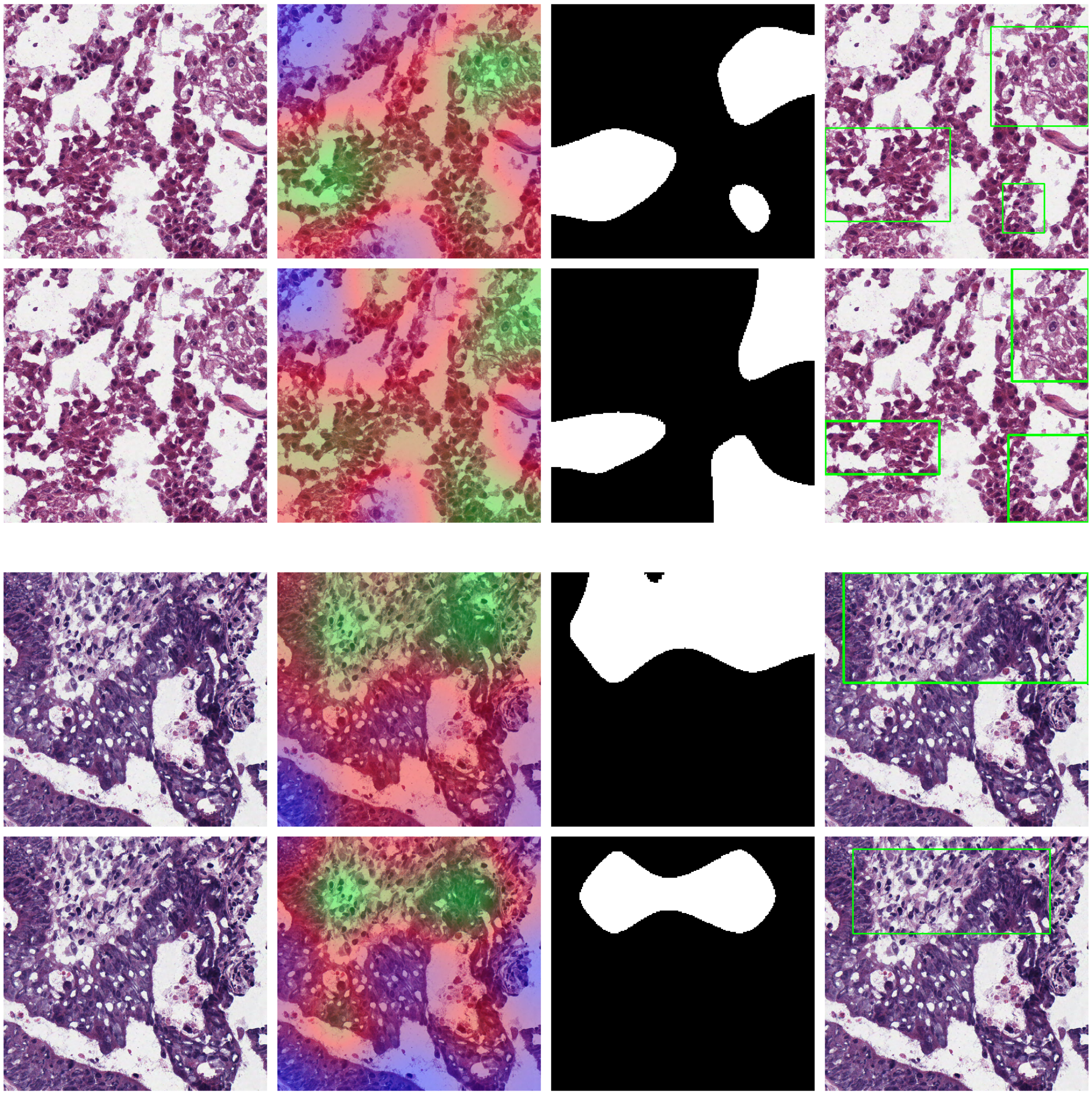

Figure S5: Cross-organ GradCAM visualizations: First and the second row shows visualization of KIRC and KICH models respectively on a sample KICH patch. Third and the fourth row shows visualization of LIHC and READ model respectively on a sample READ patch.
